# Deep Photometric Stereo Network with Multi-Scale Feature Aggregation

**DOI:** 10.3390/s20216261

**Published:** 2020-11-03

**Authors:** Chanki Yu, Sang Wook Lee

**Affiliations:** 1 Department of Media Technology, Graduate School of Media, Sogang University, Seoul 04107, Korea; ckyu@sogang.ac.kr; 2 Department of Art & Technology, School of Media, Arts and Science, Sogang University, Seoul 04107, Korea

**Keywords:** deep learning, computer vision, convolutional neural network, photometric stereo

## Abstract

We present photometric stereo algorithms robust to non-Lambertian reflection, which are based on a convolutional neural network in which surface normals of objects with complex geometry and surface reflectance are estimated from a given set of an arbitrary number of images. These images are taken from the same viewpoint under different directional illumination conditions. The proposed method focuses on surface normal estimation, where multi-scale feature aggregation is proposed to obtain a more accurate surface normal, and max pooling is adopted to obtain an intermediate order-agnostic representation in the photometric stereo scenario. The proposed multi-scale feature aggregation scheme using feature concatenation is easily incorporated into existing photometric stereo network architectures. Our experiments were performed with a DiLiGent photometric stereo benchmark dataset consisting of ten real objects, and they demonstrated that the accuracies of our calibrated and uncalibrated photometric stereo approaches were improved over those of baseline methods. In particular, our experiments also demonstrated that our uncalibrated photometric stereo outperformed the state-of-the-art method. Our work is the first to consider the multi-scale feature aggregation in photometric stereo, and we showed that our proposed multi-scale fusion scheme estimated the surface normal accurately and was beneficial to improving performance.

## 1. Introduction

3D shape reconstruction is one of the fundamental problems in computer vision. To infer the orientation of the surface, the photometric stereo exploits image irradiance variations on a surface point caused by changes of illumination direction. Over the last few decades, since it was introduced by Woodham [1] to determine the shape of an opaque material surface, substantial progress has been made in photometric stereo research. In addition, the photometric stereo technique is widely employed to recover a highly detailed 3D surface structure of an object and to inspect a visual surface defect [2,3,4,5]. In Woodham’s work, the orientation on the surface of an object is determined from a set of at least three images captured from a fixed orthographic camera under different illumination directions. This is referred to as a calibrated photometric stereo. The estimation problem of surface orientation and albedo is a linear problem for the calibrated photometric stereo with Lambertian surfaces [6] that allows it to be solved using a closed-form solution, such as a linear least-squares method. Hayakawa was the first to present the method for estimating the surface normal of Lambertian scenes when the direction of illumination is not known. This method is referred to as an uncalibrated photometric stereo, and singular value decomposition (SVD) was employed to estimate the surface normal for Lambertian scenes under directional illumination [7].

An image taken from a real scene with complex object geometry and surface reflectance is corrupted by shadows. This kind of image is also corrupted by the non-Lambertian behaviors of reflections, such as specularities and inter-reflections, that violate the Lambertian reflectance assumption. In addition, *L*_2_ norm-based solutions, such as the least-squares method and SVD, are very sensitive to outliers. In these solutions, pixels with a non-Lambertian reflection behave as outliers in a Lambertian photometric stereo. The presence of non-Lambertian reflections is the most important factor in the degradation of the quality of a surface normal that has been estimated from the photometric stereo. To overcome this problem, robust optimization-based methods, methods based on the bidirectional reflectance distribution function (BRDF), example-based methods, and deep learning-based methods have been proposed.

To alleviate the issue of violating the Lambertian assumption, some solutions using robust optimization treat the pixel value with a non-Lambertian reflection as an outlier. In these cases, a photometric stereo problem is reformulated as either an outlier-rejection problem or as an outlier-robust problem [8,9,10,11,12,13,14,15,16,17,18,19]. This approach tends to show low performance when widely spread specular reflection is observed or when the portion of observation that contains heavily non-Lambertian reflectance is very high.

The appearance of an object is determined by interactions among multiple primal components, which include viewpoint, surface structure, material, and illumination. A BRDF is widely employed to represent the surface’s appearance, and this function defines how that material interacts with light [20]. A matte object is generally represented by the Lambertian BRDF, but shiny materials exhibit more complex phenomena, such as specular reflection and multiple surface scattering. Several BRDFs have been developed to model real-world material surfaces. The use of analytical and empirical BRDF models has been popular due to their compact representations, and these models are employed to cope with the complex reflection of an object in a photometric stereo [21,22,23,24,25,26,27,28,29,30,31,32,33]. However, this approach has some drawbacks that limit their accuracy and applicability. The optimization method that is used to estimate the parameters of the BRDF and surface normal is highly unstable due to the non-convexity of complex BRDFs. Optimization can stay at a local minimum, which leads to low accuracy.

The example-based photometric stereo uses the reference objects, such as multiple types of spheres, with a homogeneous material property that is placed with target objects in the same scene [34,35,36]. This approach adopts an orientation consistency cue—the same image irradiance value is observed at two different points on the surface of objects having identical surface appearances and surface normals under the same illumination [34]. However, BRDF-based and example-based approaches cannot take cast shadows and indirect illumination effects such as inter-reflections into account, which might lead to strong biases in the recovered surface normal. The reader is referred to [37,38,39] for comprehensive surveys of traditional photometric stereo algorithms.

Deep learning algorithms have recently achieved remarkable progress in various domains, such as computer vision, speech recognition, and natural language processing. Following this trend, recent advances in the calibrated and uncalibrated photometric stereo to produce a high-fidelity surface normal have also been achieved employing deep learning, where the deep photometric stereo network learns the mapping from the multiple images to the surface normal vector [40,41,42,43,44,45,46].

In this paper, we present a convolutional neural network (CNN)-based method to discover the relationship between the surface normal and a set of an arbitrary number of images taken under a photometric stereo setup. The proposed method directly regresses on the surface normal using a fully convolutional neural network (FCN) with multi-scale feature aggregation. To the best of our knowledge, our work is the first to consider the multi-scale feature aggregation for the deep photometric stereo network. The architectures of our calibrated and uncalibrated photometric stereo network are based on the PS-FCN [41] and SDPS-Net [43], respectively. Our networks consist of feature extraction, feature aggregation, and surface normal regression branches, and the structures of the feature extraction and regression branches are similar to those of PS-FCN and SDPS-Net.

Our proposed architecture’s main contribution is the feature aggregation branch, in which the feature aggregation is obtained by the concatenation of the multi-scale feature maps, and an element-wise max-pooling operation is adopted to merge features obtained from the unordered image sequence in a permutation-invariant manner. Experiments are carried out using a DiLiGent photometric stereo benchmark dataset [39] and demonstrate that the accuracy of our proposed approach is improved over those of other photometric stereo methods based on deep learning.

The rest of this paper is organized as follows. Section 2 introduces related research on the photometric stereo and deep learning, and Section 3 presents a fully convolutional neural network with a multi-scale feature aggregation to solve calibrated and uncalibrated photometric stereo problems. Experimental results are presented in Section 4, and our conclusions are presented in Section 5.

## 2. Related Work

We review research related to the photometric stereo. The earliest photometric stereo methods only considered Lambertian surfaces [5,7]. Even though the Lambertian BRDF model is widely adopted in the photometric stereo due to its simplicity, several photometric stereo methods—the robust optimization-based, BRDF-based, example-based, and deep learning-based methods—have been proposed to cope with general materials, since the Lambertian BRDF model is not adequate for representing real-world materials.

The earliest calibrated photometric stereo methods for non-Lambertian scenes employed photometric reasoning to distinguish non-Lambertian reflections from Lambertian reflections, in a set of four images that have specular reflection and shadow. They assume that, of the four images, only one image with specular reflection and shadow is observed [8,9]. Chandraker et al. employed a graph cut-based Markov random field to detect light source visibility and to discard shadows in a set of four images [10]. The photometric stereo requires at least three images with a pure Lambertian reflection for each pixel; therefore, four images hardly suffice to find the pure Lambertian reflections for an object with a complex shape and surface appearance. These methods can be reformulated as an outlier rejection problem for distinguishing non-Lambertian reflections from Lambertian ones in an image-set that has non-Lambertian reflection components. In other words, the Lambertian reflections can be considered to be inliers, and non-Lambertian reflections and shadows can be considered to be outliers.

This outlier rejection and discounting problem is solved using several statistical and robust optimization methods for more than four images. Wu et al. presented a dense photometric stereo that employs graph-cut and tensor belief propagation algorithms and uses a large number of dense images as inputs [47]. Wu and Tang employed an expectation-maximization algorithm to estimate the albedos and surface normals from a set of dense and noisy images [11]. Verbiest and Van Gool adopted a maximum likelihood framework wherein the normal map is treated as hidden variables, and a binary inlier map is modeled as a Markov random field with an associated Gibbs prior distribution [12]. The random sample consensus (RANSAC) method is one of the most preferred and robust algorithms for estimating model parameters from data contaminated with outliers [48]. Mukaigawa et al. employed RANSAC for the calibrated photometric stereo [13]. RANSAC can be thought of as a way to minimize the *L*_0_-norm, which is the number of outliers. RANSAC does not guarantee a globally optimal solution due to this algorithm’s randomized nature. Therefore, some outliers may remain after applying RANSAC. Yu et al. presented *L*_0_-norm minimization with guaranteed global optimality for the photometric stereo, and they reformulated the calibrated photometric stereo as a maximum feasible subsystem problem to find the largest consensus set that can be satisfied with the Lambertian constraints [15]. Even when the proportion of outliers is very high, this method works well. However, *L*_0_-norm minimization is computationally expensive, so it is not adequate for application to a dense photometric stereo that deals with a large number of images. In addition, *L*_1_-norm minimization, sparse Bayesian learning, and low-rank minimization are adopted to solve the calibrated photometric stereo where non-Lambertian reflections and shadows are assumed to be sparse outliers [17,18,19]. For the uncalibrated photometric stereo, Sunkavalli et al. adopted RANSAC [14], and Miyazaki and Ikeuchi employed robust SVD to handle outliers for which the shadow and specular pixels are detected by the graph-cut algorithm [16].

Some photometric stereos use the BRDF model to handle non-Lambertian reflection. The dichromatic BRDF model that was introduced by Shafer [49] to represent the surface reflectance of dielectric objects is employed for separating or discounting specular reflections. They also take advantage of the fact that the specular reflection on the dielectric object’s surface and the illumination have the same color [21,22]. However, most of the BRDF-based approaches on the photometric stereo use a parametric BRDF model to accurately represent the image irradiance and to locally regress the diffuse albedo and non-Lambertian reflectance parameters, as well as the surface normal. They do this instead of discarding or separating non-Lambertian reflections. Micro-facet based BRDF models, including the Torrance-Sparrow model [50] and the isotropic Ward model [51], are considered for handling the reflectance property of an object’s glossy surface [23,25,26,27,52]. The bi-polynomial reflectance model has been proposed to represent the low-frequency non-Lambertian reflectance component [24]. The symmetric, monotonic, and isotropic reflectance properties of the general isotropy material are exploited in the photometric stereo [28,29,30,31,32,33]. Hertzmann and Seitz introduced an example-based method to obtain BRDF and the surface normal, which takes advantage of reference objects with a convex shape, such as spheres or cylinders [34]. The example-based approaches assume that the target BRDF can be represented by a weighted sum of several numbers of reference BRDFs. Zhuo and Sankaranarayanan presented a virtual example-based photometric stereo method to eliminate the need for physical reference objects, where the MERL BRDF dataset [53] is exploited to generate synthetic reference objects, taking the target illumination environment into account [35]. Enomoto et al. reformulated the photometric stereo problem as a discrete hypothesis-and-test search problem to a surface normal space [36].

Santo et al. proposed a deep learning-based photometric stereo, in which a fully connected neural network with a shadow layer to regress the surface normal is used in a pixel-wise manner. It is called a deep photometric stereo network (DPSN) [40]. It should be noted that a network should consider the input order of input images and an arbitrary number of input images when applying deep learning to a photometric stereo. To address this problem, Santo et al. used the image sequence captured under a set of specific illumination directions and intensities during the training and test stages. Chen et al. presented the fully convolutional neural network, called PS-FCN, where an element-wise max-pooling scheme for the photometric stereo is employed to process an unordered image sequence in a permutation invariant manner [42]. Ikehata introduced a pixel-wise observation map that was generated by a two-dimensional spherical projection of image irradiances, taking the known illumination directions into account, to obtain an intermediate order-agnostic representation from the unordered input data. This rational pseudo-invariant observation map was derived from the isotropic BRDF constraint and was used as an input for a CNN [42]. Taniai and Maehara presented an inverse rendering-based CNN architecture for a photometric stereo in an unsupervised manner. This architecture consists of a photometric stereo network to regress surface normals and an image reconstruction network to reconstruct the observed images in an inverse rendering manner [45]. For a sparse photometric stereo, Li et al. introduced a trainable two-dimensional occlusion layer to manage cast shadows in the CNN framework [44], and Zheng et al. presented a lighting interpolation network to yield a dense observation map from a sparse set of input and illumination pairs [46]. Chen et al. presented a two-stage deep learning architecture, called a self-calibrating photometric stereo network (SDPS-Net), consisting of a lighting calibration network (LCNet) and a normal estimation network (NENet). The LCNet regresses an illumination direction and intensity per image. The NENet regresses a surface normal, in which the illumination information is estimated from the LCNet, and a set of input images is used as input for NENet [43].

## 3. Fully Convolutional Neural Network with a Multi-Scale Feature Aggregation

In this paper, we present an image formation model and a fully convolutional neural network with multi-scale feature aggregation to solve calibrated and uncalibrated photometric stereo problems where the element-wise max-pooling scheme is adopted to obtain an order-agnostic intermediate representation.

### 3.1. Image Formation Model

We are interested in the problem of estimating surface normals using the CNN-based photometric stereo, where it assumes that the camera follows an orthographic projection model with a linear radiometric response, and illumination follows a directional lighting model. Consider a surface point with surface normal ***n***∈**ℜ**^3^ illuminated by an incoming directional light source. This illumination source is defined by the light intensity *e*∈**ℜ**^+^ and the light direction ***l***∈**ℜ**^3^. When a non-Lambertian scene is observed from viewing direction ***v***∈**ℜ**^3^, the observed intensity *I*∈**ℜ**^+^ at a pixel is defined as

(1) $$I\;=\;e\rho \left(l,n,v\right)\max\left(n^{T}l,\;0\right)\;+\varepsilon ,$$

where ***l***, ***n***, and ***v*** are a unit vector. *ρ*(***l***,***n***,***v***) is a general isotropic BRDF to represent the surface appearance of isotropic materials and this function is dependent on the light direction, surface normal, and viewing direction. ***n****^T^**l*** is the cosine of the angle between the surface normal and the light direction. A max function is added to account for an attached shadow, which returns the larger value between ***n****^T^**l*** and zero.***ε*** denotes the noise, outlier, cast shadow, and indirect illumination effects, including inter-reflection.

Given a set of images captured at a fixed viewpoint of the viewing direction ***v*** = [0, 0, 1]*^T^* with corresponding distant illuminations of light intensities ***E*** = [*e*_1_ *e*_2_ … *e_k_*]*^T^*∈**ℜ***^k^* and light directions ***L*** = [***l***_1_ ***l***_2_ … ***l****_k_*]*^T^*∈**ℜ***^k^*^×3^, the CNN architecture to apply photometric stereo should be both invariant to the order of input images and capable of processing a variable-length input image sequence. In addition, order-agnostic operations such as element-wise max or average pooling schemes in CNN have been proposed to combine deep features extracted from an arbitrary number of input images in computer vision problems such as photometric stereo, multi-view stereo, and burst image [41,43,54,55,56]. Our proposed CNNs map a series of unordered images to a surface normal map in a permutation-invariant manner, in which a max-pooling operation is adopted to merge information extracted from multiple unordered images.

### 3.2. Calibrated Photometric Stereo Network

**Figure 1:**
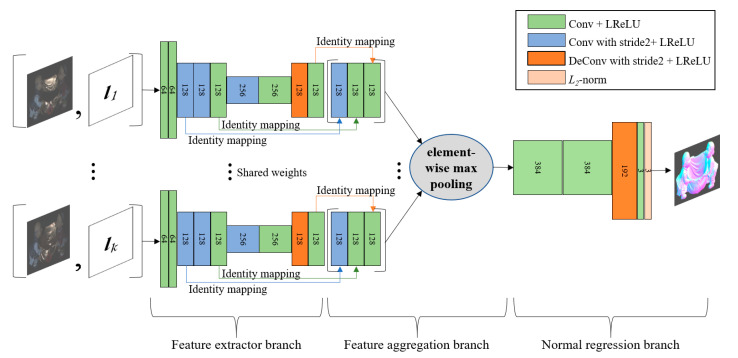
Network architecture for calibrated photometric stereo. The numbers in layers depict the number of output channels.

Figure 1 illustrates the network architecture for calibrated photometric stereo to regress a surface normal map. Our model’s structure is based on PS-FCN [41] and then modified for aggregating multi-scale features using feature concatenation. Our network consists of feature extractor branches, a feature aggregation branch, and a normal regression branch.

Each image, divided by its corresponding light intensity and the direction vector of its corresponding light, is fed into a feature extractor branch. In a feature extractor branch of Figure 1, the input with six-dimensional arrays of fixed-size was < *H* × *W* >, where *H* and *W* are the spatial dimensions, is passed through a stack of convolution and deconvolution layers with kernel size < *h* × *w* × *d* > where *h* and *w* are the spatial dimensions and *d* is the number of output channels in a kernel. In addition, each convolution or deconvolution layer is followed by a leaky rectified linear unit (LReLU) activation function, and the convolution layer with a stride of 2 produces output feature maps that have half the resolution of the input feature maps. In addition, multiple feature extractor branches share the same weights.

The first step in the feature aggregation branch is to extract a local intermediate representation of size < *H*/2 × *W*/2 × 384 > from each feature extractor branch, which is produced by concatenating feature maps created at three different scales in a feature extraction branch. In general, different-scale features are combined into the same-resolution features through an up-sampling process, a down-sampling process, and a skip connection; The representations for three different scale resolution (e.g., the feature maps of size < *H* × *W* × 64 >, size < *H*/2 × *W/*2 × 128 > and size < *H*/4 × *W/*4 × 256 > for the second, fifth, and seventh convolutional blocks) may be used to obtain a fused representation in our network architecture. However, the feature map of size < *H*/2 × *W/*2 × 128 > for the third, fifth, and ninth convolutional blocks are alternatively used to create a local intermediate representation with the same spatial size of the feature map, as seen in Figure 1; The local intermediate representation of size < *H*/2 × *W/*2 × 384 > for each input is achieved through identity skip connections and a concatenation operation, which has an advantage of avoiding the additional computational cost of the up-sampling and the down-sampling processes. At the second step in the feature aggregation branch, all local intermediate representations with respect to the input pairs of image and illumination are aggregated into a single global intermediate representation by an element-wise max-pooling operation to obtain order-agnostic features from a variable number of unordered inputs. In addition, the element-wise max-pooling operation calculates the largest value in each position over all local intermediate representations; therefore, the local and global intermediate representations have the same size.

The global intermediate representation is fed into a regression branch that consists of three convolutional layers, a deconvolutional layer, and an *L*_2_ normalization layer. The output of the last deconvolutional layer is normalized using an *L*_2_ normalization layer to ensure the unit vector at each pixel of the surface normal map of size < *H* × *W* × 3 >. In addition, the spatial dimensions of kernels of all convolutional layers used in our networks are < 3 × 3 > and those of the deconvolutional layers are < 4 × 4 > with a stride of 2; All the convolutional layers except the last one include a bias term, on the other hand, all the deconvolutional layers do not include the bias term.

### 3.3. Uncalibrated Photometric Stereo Network

**Figure 2:**
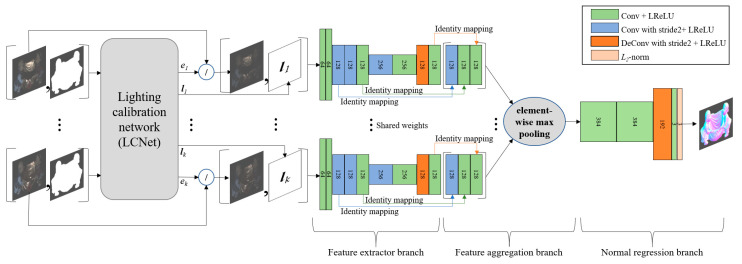
Network architecture for uncalibrated photometric stereo. The numbers in layers depict the number of output channels.

Figure 2 illustrates the network architecture for an uncalibrated photometric stereo to regress a surface normal map. Our model’s structure is based on an SDPS-Net that consists of two sub-networks: an LCNet for inferring the illumination information and a NENet for regressing the surface normal [43]. Our proposed network uses the LCNet of SDPS-Net without any modification and employs the NENet presented in Section 3.2, and the input layer of the proposed NENet is modified to exploit the light information estimated with the LCNet.

The inputs of the LCNet are formed by concatenating each color image of size < *H* × *W* × 3 > with its corresponding mask image of size < *H* × *W* × 1 > where the LCNet infers the light intensity and direction for each image. It should be noted that an object-mask image is only used in the LCNet. For surface normal estimation, inputs are formed by concatenating each color image divided by the intensity of its corresponding light estimated by the LCNet, with the light direction estimated by the LCNet fed into the proposed NENet. In addition, the size of the input for the proposed NENet is < *H* × *W* × 6 > for each input and the size of the final output is < *H* × *W* × 3 > for the proposed NENet.

### 3.4. Loss Function and Training Data

Cosine distance, which measures the angles between the predicted and ground-truth normal vector, is adopted as a loss function of the proposed NENet described in Section 3.2 and Section 3.3 This loss function is defined as follows:

(2) $$L\left(n_{i},n_{i}^{gt}\right)=\sum_{i}^{M}\left(1-\cos\left(n_{i},n_{i}^{gt}\right)\right)$$

where ***n****_i_* and $n_{i}^{gt}$ denote a predicted surface normal and the ground-truth of a surface normal at the *i^th^* pixel, respectively, and *M* indicates the number of pixels. The Adam optimization method [57] was exploited to minimize the loss function of (2).

Capturing the ground-truth surface normals of real-world scenes with complex shapes and spatially varying materials at a large scale is extremely challenging. To mitigate this challenge, recent works have adopted a method of training networks on large-scale and physically meaningful synthetic datasets as an alternate way [40,41,42,43,44,45,46]. The proposed NENets are trained on publicly available synthetic photometric stereo datasets used in the training procedure of PS-FCN and SDPS-Net. These datasets are generated using Mitsuba [58], which is an open-source physically based renderer where a Blob Shape Dataset [59] and a Sculpture Shape Dataset [55] contribute to providing the shape information, including the surface normal, as well as 100 isotropic BRDFs from the MERL BRDF dataset that contribute to representing a variety of real-world materials. In addition, cast shadows and inter-reflections are considered in the image rendering procedure, and these datasets consist of 5,453,568 color images of spatial dimensions < 128 × 128 >.

Although our NENets described in Section 3.2 and Section 3.3. have the same structure, the NENet for the calibrated photometric stereo uses accurate calibrated light information, while the NENet for the uncalibrated photometric stereo uses inaccurate light information estimated with the pre-trained LCNet. Hence, the two NENet are independently trained with different hyper-parameters.

## 4. Experimental Results

We conducted experiments on the calibrated and uncalibrated photometric stereo evaluations to demonstrate the proposed algorithm’s accuracy and evaluate our algorithm on the DiLiGent photometric stereo benchmark dataset [39]. This dataset consists of ten real-world objects where 96 images captured under the calibrated light conditions, an object-mask image, and the ground-truth surface normal are given for each object. Our method is implemented in PyTorch [60], and all experiments were performed on a Windows server machine with Nvidia Tesla V100 PCIe 32 GB GPU and 128 GB RAM.

Most of the hyper-parameters setting of the proposed NENets for the calibrated and uncalibrated photometric stereo follow those of PS-FCN and SDPS-Net, respectively. Our NENet for calibrated photometric stereo was trained from scratch for 40 epochs, and the Pytorch Adam optimizer with default parameters except a learning rate was exploited to optimize the parameters, where the initial value of the learning rate was set to 0.001, and this rate was decayed by a factor of 0.5 every 5 epochs. The batch size in the training and validation procedures is set to 32 and 8, respectively. Color images of a spatial size < 32 × 32 > randomly cropped from the given color images of the spatial size < 128 × 128 > were used as inputs during training. We did not carry out image geometric transformation, color augmentation, and noise addition for the training procedure. In addition, our NENet for uncalibrated photometric stereo was trained from scratch for 12 epochs, and the initial value of the learning rate was set to 0.005. The learning rate was further divided by a factor of 0.5 every 2 epochs. The batch sizes in the training and validation procedures were set to 20 and 12, respectively. During training, color images of the spatial size < 128 × 128 > were used as inputs without applying any data augmentation methods.

### 4.1. Comparision with the Baseline Model

**Figure 3:**
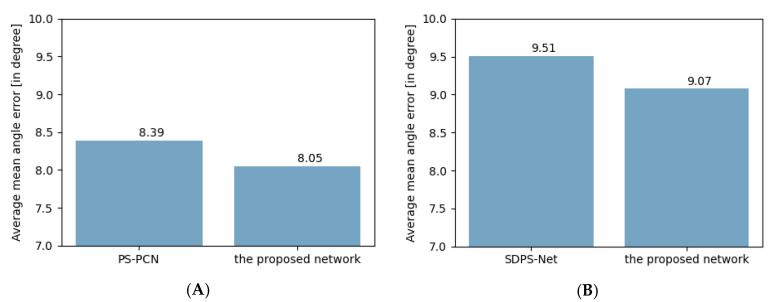
The accuracy comparison ours with baseline models: (**A**) calibrated photometric stereo evaluation; (**B**) uncalibrated photometric stereo evaluation

To show the proposed multi-scale feature aggregation scheme’s effectiveness in the photometric stereo network, we compared our model with the baseline models, PS-FCN and SDPS-Net, as briefly reported in Figure 3.

**Figure 4:**
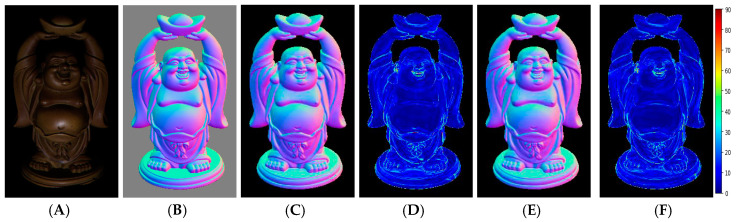
Visual quality comparisons on the Buddha object in the DiLiGent benchmark for the calibrated photometric stereo: (**A**) one image among 96 input images; (**B**) ground-truth surface normal map; (**C**) surface normal map estimated with PS-FCN; (**D**) angular error map for PS-FCN; (**E**) surface normal map estimated with the proposed method; (**F**) angular error map for the proposed method.

For the calibrated photometric stereo experiment, the predicted surface normal with the proposed method achieved an average mean angular error (MAE) of 8.05° on the DiLiGent dataset, and this proposed method surpassed the performance of PS-FCN that obtained an average MAE of 8.39°. In addition, the results of the proposed method show that the prediction accuracy was improved in eight objects of the DiLiGent dataset compared to those of the PS-FCN, as seen in Table 1**.** Figure 4 shows visual quality comparisons on the Buddha object of the DiLiGenT benchmark for PS-FCN and the proposed calibrated photometric stereo method. Figure 4A shows one image among input images used by our network and PS-FCN. Figure 4B shows the color-encoded map of the ground-truth surface normals for the Buddha object corresponding to the image shown in (a), and this color map was obtained by RGB encoding: [R, G, B] = ([*n_x_*, *n_y_*, *n_z_*] + 1)/2 where [*n_x_*, *n_y_*, *n_z_*] depicts a unit vector of the surface normal. In other words, the values in this color map were converted from [−1, 1] to [0, 1] per channel to encode the surface normal vector at each pixel, and it should be noted that this encoded color map was only used to visualize the experimental results. Figure 4C shows a color-encoded map of surface normals estimated with PS-FCN. Figure 4D shows an angular error map of surface normals estimated with PS-FCN where a heatmap is used to represent the angular error with respect to the ground truth per pixel for visualization purposes. The color value in this heatmap depicts the angular error value ranging from 0°–90° as seen in the color bar on the right side of Figure 4F. Figure 4E shows a color-encoded surface normal map for the proposed method. Figure 4F shows an angular error map of surface normals estimated with the proposed method. Please refer to Figure S1 for more results.

**Figure 5:**
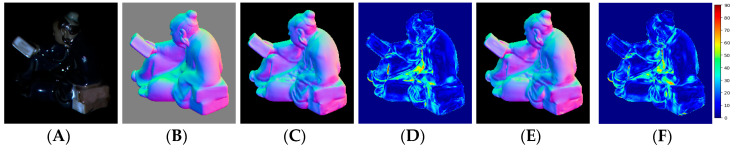
Visual quality comparisons on the Reading object in the DiLiGent benchmark for the uncalibrated photometric stereo: (**A**) one image among 96 input images; (**B**) ground-truth surface normal map; (**C**) surface normal map estimated with SDPS-Net; (**D**) angular error map for SDPS-Net; (**E**) surface normal map estimated with the proposed method; (**F**) angular error map for the proposed method.

For the uncalibrated photometric stereo experiment, the predicted surface normal with the proposed method and SDPS-Net achieved average MAEs of 9.07° and 9.51°, respectively. In addition, the results of the proposed method showed that the prediction accuracy was improved over the SDPS-Net in nine out of ten objects in the DiLiGent dataset, as seen in Table 2. Figure 5 shows visual comparisons with the Harvest object of the DiLiGenT benchmark for the SDPS-Net and the proposed uncalibrated photometric stereo method where (A) shows one image among input images, (B) shows the color-encoded map of ground-truth surface normals, (C) and (E) show a color-encoded surface normal map for SDPS-Net and the proposed method, respectively, (D) and (F) show an angular error map of surface normals estimated with SDPS-Net and the proposed method, respectively. Please refer to Figure S2 for more results.

In our experiment, we observed the degradation of the model’s prediction accuracy when a normalization layer, such as batch, instance, weight, and group normalization was added to convolutional or deconvolutional blocks; The degraded performance of the model was also observed when 1 × 1 or 3 × 3 convolution is used in the feature aggregation branch after applying concatenation. In addition, the degradation of the performance was also observed when the skip connection layer with a skip connection and a 1 × 1 or a 3 × 3 convolutional layer was used instead of the identity skip connection.

Our results showed that our proposed multi-scale fusion scheme in the photometric stereo network was beneficial for improving the accuracy of the surface normal estimation. In particular, the existing methods tend to severely degrade the estimation accuracy of the surface normal in the non-convex region where the surface reflectance of a non-convex object cannot be defined in BRDF due to global illumination effects such as a multiple surface scattering, and severe cast shadows due to complex geometry are also observed. Our results using multi-scale feature aggregation show that the estimation accuracy is particularly improved in non-convex regions over previous methods.

### 4.2. Comparision with Other Models

We compared our model with the existing method. Table 1 shows quantitative comparisons on the DiLiGent benchmark dataset in which our method was compared with previous work on the calibrated photometric stereo in terms of MAE where the numbers depict the MAE between the estimation and the ground truth of the surface normal. Our method shows the third-best performance out of thirteen methods where the proposed method, CNN-PS, PS-FCN, TM18, and DPSN are deep learning (DL)-based approaches, and HS17, EW20, ST14, IA14, GC10, AZ08, WG10, and Least Squares belong to non-DL-based approach. In addition, CNN-PS, HS17, DPSN, EW20, ST14, IA14, GC10, AZ08, WG10, and least squares were used to estimate the surface normal in a pixel-wise fashion. However, the proposed method, PS-FCN, and TM18 were performed in an image-wise fashion. CNN-PS and HS17 showed the best and second-best performances in terms of accuracy; however, their computational efficiency was low. The proposed method shows the best performance over all the existing methods for one object (Buddha) out of ten in the DiLiGent dataset and shows the second-best and third-best performances for the two objects (Cow and Harvest) and one object (Pot2), respectively. In addition, the proposed method shows the best performance among methods that were performed in an image-wise fashion.

For the uncalibrated photometric stereo, Table 2 shows quantitative comparisons on the DiLiGent benchmark dataset; Here, our method shows the best performance out of the nine methods. The proposed method, SDPS-Net, and UPS-FCN are DL-based approaches that were performed in an image-wise fashion. LC18, PF14, WT13, LM13, SM10, and AM07 belong to the non-DL-based approaches that were performed in a pixel-wise fashion. In addition, the proposed method shows better performance than all existing methods for nine objects (Ball, Pot1, Bear, Pot2, Buddha, Goblet, Reading, Cow, and Harvest) out of ten in the DiLiGent dataset, and shows the second-best performance for the Cat object.

## 5. Conclusions

This paper proposed a photometric stereo method based on the convolutional neural network with a multi-scale feature aggregation scheme. Our experiments demonstrated that our calibrated photometric stereo approach obtains comparable performance to state-of-the-art methods, and our uncalibrated photometric stereo approach outperformed the state-of-the-art method. This shows that aggregating multi-scale feature maps in photometric stereo help improve the performance of the surface normal estimation.

The surface appearance is determined by the interaction among the surface normal, material, and lighting. Nevertheless, our model only learns the surface normal from the image. Hence, future research should include the development of simultaneous estimations of the surface normal and BRDF, including diffuse and specular components.

## Figures and Tables

**Table 1:** Accuracy comparison for the DiLiGenT benchmark in the surface normal estimation of calibrated photometric stereo methods. The value indicates the mean angular errors in degrees for estimated surface normal where red, blue, and green indicate the best, second-best, and third-best performances, respectively.

Method	Ball	Cat	Pot1	Bear	Pot2	Buddha	Goblet	Reading	Cow	Harvest	Average
Ours	2.84	5.71	6.65	6.87	**7.03**	**7.88**	8.67	12.85	**6.39**	**15.62**	**8.05**
CNN-PS [42]	2.20	**4.60**	**5.40**	**4.10**	**6.00**	**7.90**	**7.30**	**12.60**	8.00	**14.00**	**7.20**
HS17 [35]	**1.33**	**4.88**	**5.16**	**5.58**	**6.41**	8.48	**7.57**	**12.08**	8.23	**15.81**	**7.55**
PS-FCN [41]	2.82	6.16	7.13	7.55	7.25	**7.91**	**8.60**	13.33	**7.33**	15.85	8.39
TM18 [45]	**1.47**	**5.44**	**6.09**	**5.79**	7.76	10.36	11.47	**11.03**	**6.32**	22.59	8.83
DPSN [40]	2.02	6.54	7.05	6.31	7.86	12.68	11.28	15.51	8.01	16.86	9.41
EW20 [36]	**1.58**	6.30	6.67	6.38	7.26	13.69	11.42	15.49	7.80	18.74	9.53
ST14 [24]	1.74	6.12	6.51	6.12	8.78	10.60	10.09	13.63	13.93	25.44	10.30
IA14 [29]	3.34	6.74	6.64	7.11	8.77	10.47	9.71	14.19	13.05	25.95	10.60
GC10 [52]	3.21	8.22	8.53	6.62	7.90	14.85	14.22	19.07	9.55	27.84	12.00
AZ08 [31]	2.71	6.53	7.23	5.96	11.03	12.54	13.93	14.17	21.48	30.50	12.61
WG10 [17]	2.06	6.73	7.18	6.50	13.12	10.91	15.70	15.39	25.89	30.01	13.35
Least Squares [1]	4.10	8.41	8.89	8.39	14.65	14.92	18.50	19.80	25.60	30.62	15.39

**Table 2:** Accuracy comparison for the DiLiGenT benchmark in the surface normals estimation of uncalibrated photometric stereo methods. the value indicates the mean angular errors in degrees for estimated surface normals where red, blue, and green indicate the best, second-best, and third-best performances, respectively.

Method	Ball	Cat	Pot1	Bear	Pot2	Buddha	Goblet	Reading	Cow	Harvest	Average
Ours	**2.75**	**9.35**	**7.73**	**5.45**	**6.96**	**8.88**	**11.24**	**14.87**	**6.72**	**16.77**	**9.07**
SDPS-Net [43]	**2.77**	**8.06**	**8.14**	**6.89**	**7.50**	**8.97**	**11.91**	**14.90**	**8.48**	**17.43**	**9.51**
UPS-FCN [41]	6.62	14.68	13.98	11.23	**14.19**	15.87	20.72	23.26	**11.91**	**27.79**	**16.02**
LC18 [33]	9.30	12.60	12.40	10.90	15.70	19.00	**18.30**	**22.30**	15.00	28.00	16.30
PF14 [61]	4.77	**9.54**	9.51	9.07	15.90	14.92	29.93	24.18	19.53	29.21	16.66
WT13 [28]	**4.39**	36.55	**9.39**	**6.42**	14.52	**13.19**	20.57	58.96	19.75	55.51	23.93
LM13 [62]	22.43	25.01	32.82	15.44	20.57	25.76	29.16	48.16	22.53	34.45	27.63
SM10 [63]	8.90	19.84	16.68	11.98	50.68	15.54	48.79	26.93	22.73	73.86	29.59
AM07 [64]	7.27	31.45	18.37	16.81	49.16	32.81	46.54	53.65	54.72	61.70	37.25

## Supplementary Materials

The following are available online at https://www.mdpi.com/1424-8220/20/21/6261/s1, Figure S1. Visual quality comparisons on the Ball, Cat, Pot1, Bear, Pot2, Globet, Reading, Cow, and Harvest objects in the DiLiGent benchmark for the calibrated photometric stereo: (the first column) one image among 96 input images; (the second column) ground-truth surface normal map; (the third column) surface normal map estimated with PS-FCN; (the fourth column) angular error map for PS-FCN; (the fifth column) surface normal map estimated with the proposed method; (the sixth column) angular error map for the proposed method. Figure S2. Visual quality comparisons on the Ball, Cat, Pot1, Bear, Pot2, Buddha, Goblet, Cow, and Harvest object in the DiLiGent benchmark for the uncalibrated photometric stereo: (the first column) one image among 96 input images; (the second column) ground-truth surface normal map; (the third column) surface normal map estimated with SDPS-Net; (the fourth column) angular error map for SDPS-Net; (the fifth column) surface normal map estimated with the proposed method; (the sixth column) angular error map for the proposed method.
