# Supplementary material for: Deep Photometric Stereo Network with Multi-Scale Feature Aggregation
Source: Sensors (Basel). 2020 Nov 3;20(21):6261. doi: 10.3390/s20216261 (PMC7675179; doi:10.3390/s20216261)
Supplement: Supplementary file 1 [file sensors-20-06261-s001.pdf]

sensors

Article

Deep Photometric Stereo Network with Multi-Scale Feature Aggregation

Chanki Yu [1] and Sang Wook Lee [1,2,*]

[1] Department of Media Technology, Graduate School of Media, Sogang University, Seoul 04107 Korea; ckyu@sogang.ac.kr (C.Y.); slee@sogang.ac.kr (S.W.L.)
[2] Department of Art & Technology, School of Media, Arts and Science, Sogang University, Seoul 04107, Korea
* Correspondence: slee@sogang.ac.kr; Tel.: +82-2-705-8902

Received: 8 October 2020; Accepted: 2 November 2020; Published: 3 November 2020

Supplementary Materials:

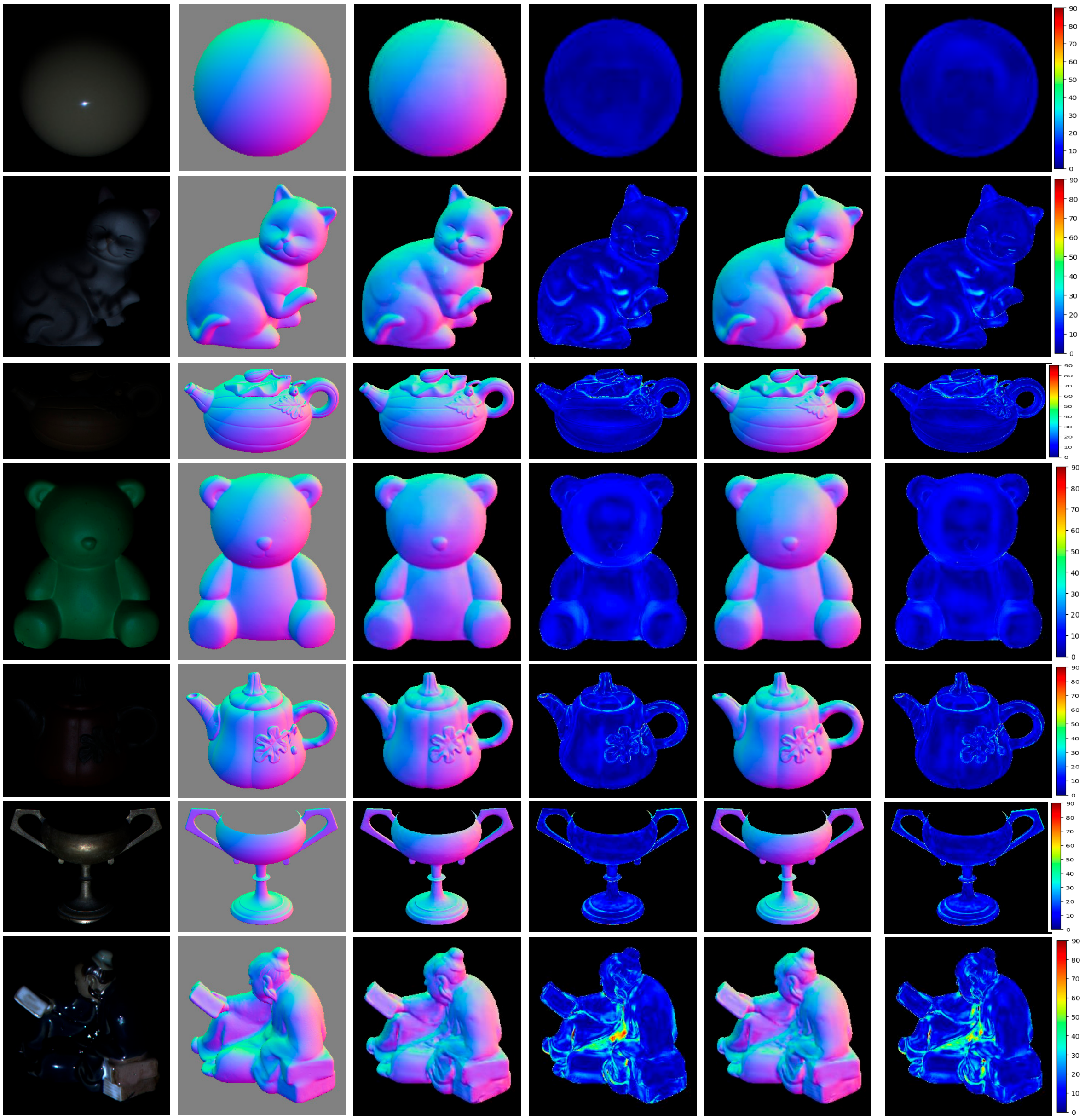

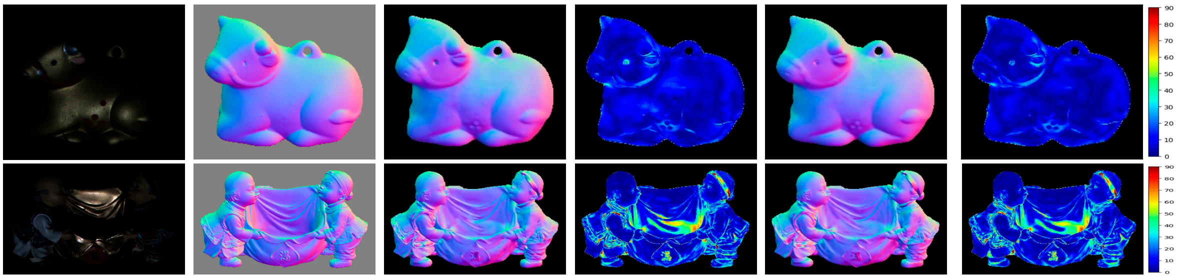

Figure S1. Visual quality comparisons on the Ball, Cat, Pot1, Bear, Pot2, Globet, Reading, Cow, and Harvest objects in the DiLiGent benchmark for the calibrated photometric stereo: (the first column) one image among 96 input images; (the second column) ground-truth surface normal map; (the third column) surface normal map estimated with PS-FCN; (the fourth column) angular error map for PS-FCN; (the fifth column) surface normal map estimated with the proposed method; (the sixth column) angular error map for the proposed method.

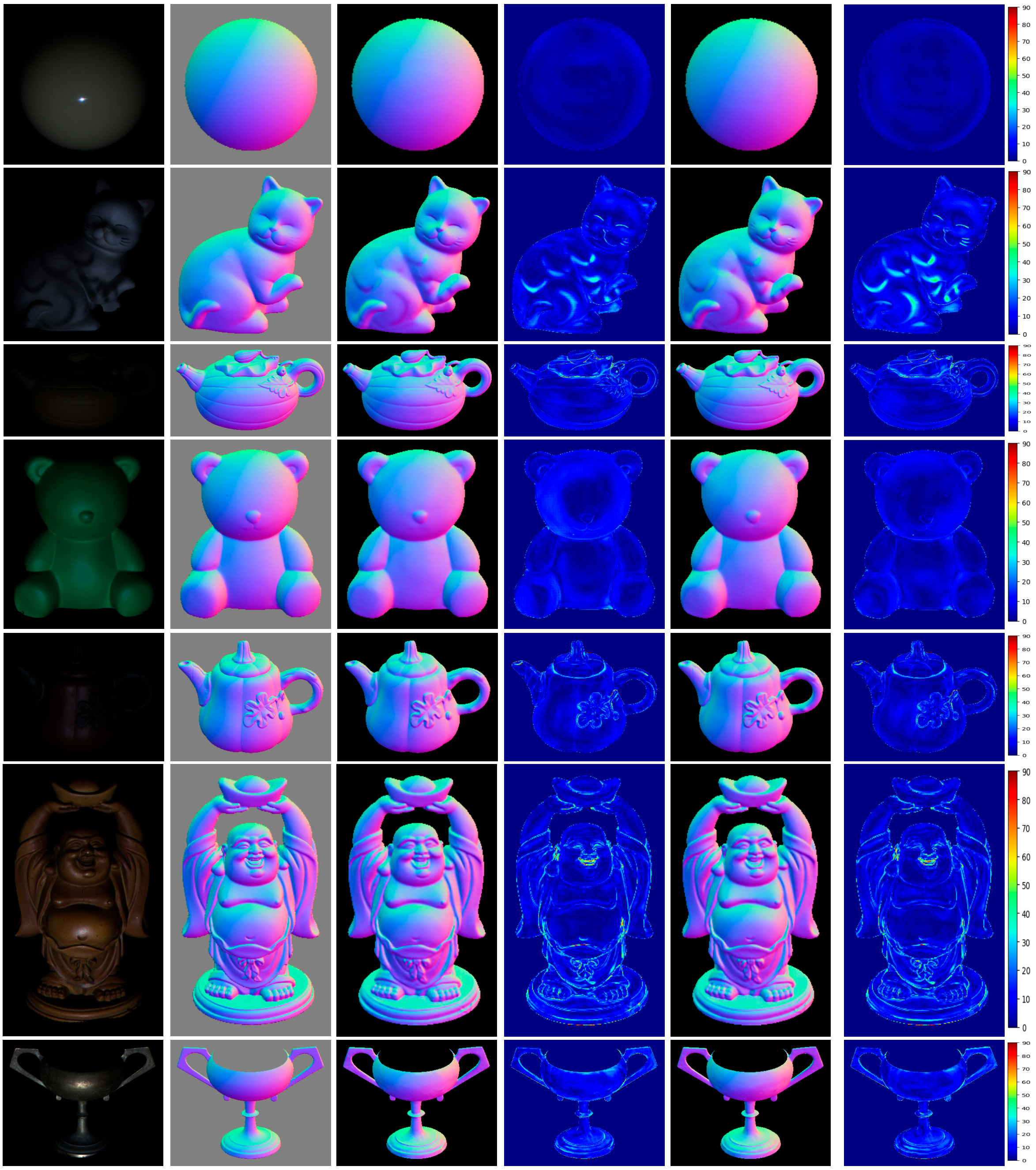

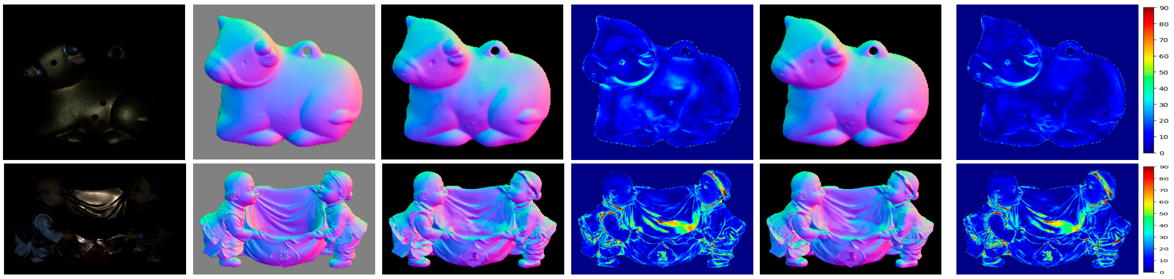

Figure S2. Visual quality comparisons on the Ball, Cat, Pot1, Bear, Pot2, Buddha, Goblet, Cow, and Harvest object in the DiLiGent benchmark for the uncalibrated photometric stereo: (the first column) one image among 96 input images; (the second column) ground-truth surface normal map; (the third column) surface normal map estimated with SDPS-Net; (the fourth column) angular error map for SDPS-Net; (the fifth column) surface normal map estimated with the proposed method; (the sixth column) angular error map for the proposed method.

© 2020 by the authors. Submitted for possible open access publication under the terms and conditions of the Creative Commons Attribution (CC BY) license (http://creativecommons.org/licenses/by/4.0/).
